# *tert*-Butyl 2-{[5-(4-cyano­phen­yl)pyridin-3-yl]sulfon­yl}acetate

**DOI:** 10.1107/S160053681400511X

**Published:** 2014-03-12

**Authors:** H. C. Devarajegowda, B. S. Palakshamurthy, K. E. Manojkumar, S. Sreenivasa

**Affiliations:** a Department of Physics, Yuvaraja’s College (Constituent College), University of Mysore, Karnataka, India; b Department of Studies and Research in Chemistry, Tumkur University, Tumkur, Karnataka 572 103, India

## Abstract

In the title compound, C_18_H_18_N_2_O_4_S, the dihedral angle between the aromatic rings is 33.71 (9)° and an intra­molecular C—H⋯O hydrogen bond closes an *S*(6) ring. In the crystal, mol­ecules are linked by C—H⋯O and C—H⋯N hydrogen bonds to generate a three-dimensional network. A very weak aromatic π–π stacking inter­ction is also observed [centroid–centroid separation = 3.9524 (10) Å].

## Related literature   

For the biological activity of nitro­gen-containing heterocylces, see: Demirbas *et al.* (2005 ▶); Manojkumar *et al.* (2013 ▶).
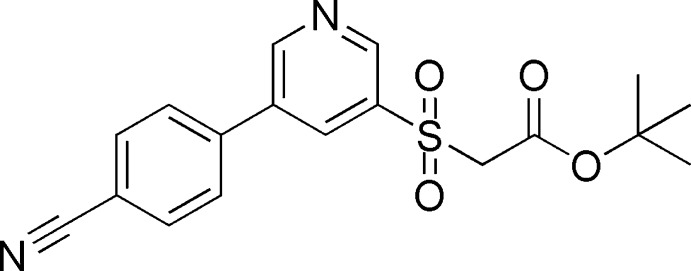


## Experimental   

### 

#### Crystal data  

C_18_H_18_N_2_O_4_S
*M* *_r_* = 358.40
Monoclinic, 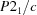

*a* = 17.3871 (7) Å
*b* = 12.5318 (5) Å
*c* = 8.4297 (3) Å
β = 99.103 (2)°
*V* = 1813.63 (12) Å^3^
*Z* = 4
Mo *K*α radiation
μ = 0.20 mm^−1^
*T* = 294 K
0.36 × 0.28 × 0.22 mm

#### Data collection  

Bruker APEXII CCD diffractometer
Absorption correction: multi-scan (*SADABS*; Bruker, 2009 ▶) *T*_min_ = 0.931, *T*_max_ = 0.957
13884 measured reflections
3176 independent reflections
2649 reflections with *I* > 2σ(*I*)
*R*_int_ = 0.029

#### Refinement  

*R*[*F* ^2^ > 2σ(*F* ^2^)] = 0.038
*wR*(*F* ^2^) = 0.113
*S* = 1.07
3176 reflections
229 parameters
H-atom parameters constrained
Δρ_max_ = 0.23 e Å^−3^
Δρ_min_ = −0.30 e Å^−3^

### 

Data collection: *APEX2* (Bruker, 2009 ▶); cell refinement: *SAINT-Plus* (Bruker, 2009 ▶); data reduction: *SAINT-Plus*; program(s) used to solve structure: *SHELXS97* (Sheldrick, 2008 ▶); program(s) used to refine structure: *SHELXL97* (Sheldrick, 2008 ▶); molecular graphics: *ORTEP-3 for Windows* (Farrugia, 2012 ▶) and *Mercury* (Macrae *et al.*, 2008 ▶); software used to prepare material for publication: *SHELXL97*.

## Supplementary Material

Crystal structure: contains datablock(s) I, New_Global_Publ_Block. DOI: 10.1107/S160053681400511X/hb7202sup1.cif

Structure factors: contains datablock(s) I. DOI: 10.1107/S160053681400511X/hb7202Isup2.hkl

Supporting information file. DOI: 10.1107/S160053681400511X/hb7202Isup3.cml

CCDC reference: 985495

Additional supporting information:  crystallographic information; 3D view; checkCIF report

## Figures and Tables

**Table 1:** Hydrogen-bond geometry (Å, °)

*D*—H⋯*A*	*D*—H	H⋯*A*	*D*⋯*A*	*D*—H⋯*A*
C9—H9⋯O1^i^	0.93	2.50	3.210 (2)	134
C12—H12⋯N2^ii^	0.93	2.60	3.518 (2)	172
C13—H13*A*⋯O1^iii^	0.97	2.54	3.347 (2)	141
C16—H16*A*⋯O4	0.96	2.36	2.971 (4)	121

Symmetry codes: (i) 
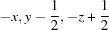
; (ii) 
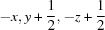
; (iii) 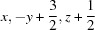.

**Fig. 1.**
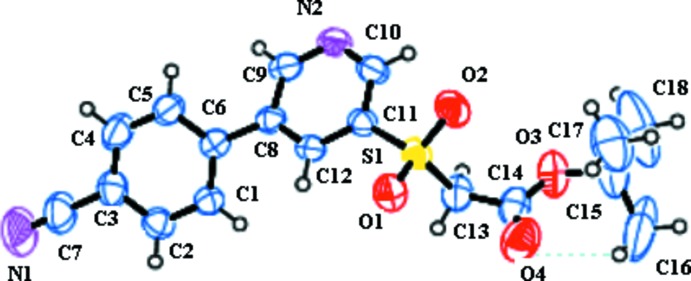
Molecular structure of the title compound, showing displacement ellipsoids drawn at the 50% probability level.

**Fig. 2.**
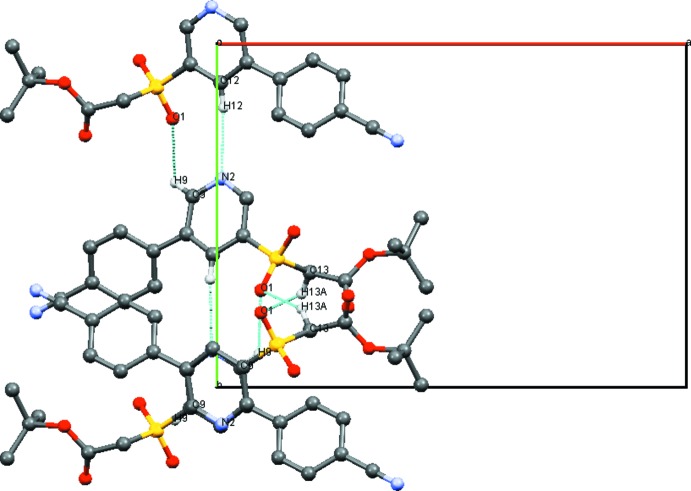
Packing of the molecule.

**Fig. 3.**
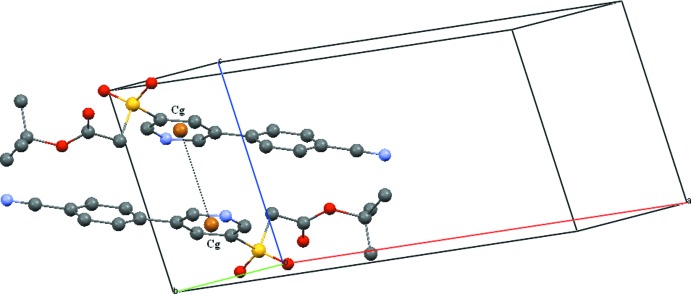
The molecule with π-π stacking.

## supplementary crystallographic information

### 1. Comment

Nitrogen containing heterocyclic molecules show properties like Antibacterial, Anthelmintic and Anti-Inflammatory Agents antifungal [Manojkumar *et al.*, 2013; Demirbas *et al.*, 2005] *etc*. In perticular, 4-pyridin-3-yl benzonitrile nucleus has been the focus of our recent research related to design liquid crystals (our unpublished results). Keeping this in mind the title compound was synthesized and its crystal structure determined.

In the title structure, C_18_H_18_N_2_O_4_S, the dihedral angle between benzene and pyridine ring is 33.71 (9)°. and an intramolecular C16—H16A···O4 hydrogen bond closes an *S*(6) ring. The crystal structure displays C9—H9···O1, C13—H13A···O1 and C12—H12···N2 hydrogen bonding forming C(7), C(4) and C(5) chains along [010], [001] and [010] respectively. A weak aromatic π-π stacking interaction is also observed [centroid-centroid separation = 3.9524 (10) Å].

### 2. Experimental

5-(Methylsulfonyl)pyridin-3-ylboronic acid (1 mol) was taken in 1,4-dioxane and water (60:40 ml). Bis(triphenylphosphine)palladium(II) dichloride (dikis) (0.03 mol) and K_2_CO_3_ (3 mol) were added to the above solution. The solvent was degassed with argon for one hour and 4-iodobenzonitrile (1 mol) was added. Heated the contents for 5 h. The reaction was monitored by TLC, filtered the reaction mixture by using celite and the solvent was removed by rota evaporator. The crude compound was purified by 60–120 silica gel column chromatography to yield pure yellow solid of 4-(5-(methylsulfonyl)pyridin-3-yl)benzonitrile.

4-(5-(methylsulfonyl)pyridin-3-yl)benzonitrile (1 mol) was taken in dry THF (25 ml) and to this Lithium Hexamethyldisilazide (LiHMDS) in THF (1.5 mol) and Boc anhydride were added. The. reaction mixture was stirred at room temperature for 5 h and completion of the reaction was confirmed by TLC. The reaction mixture was quenched by ice cold water and later extracted with ethyl acetate. Solvent was dried over anhydrous sodium sulfate and concentrated under vacuum to give crude product. The crude Product obtained was purified by column chromatography to get pure *tert*-butyl 2-(5-(4-cyanophenyl)pyridin-3-ylsulfonyl)acetate, which was recrystallized by dichloromethane and methanol (9:1) system to yield colourless prisms.

### 3. Refinement

The H atoms were positioned with idealized geometry using a riding model with C—H = 0.93–0.97 Å. All H atoms were refined with isotropic displacement parameters (set to 1.2–1.5 times of the U eq of the parent atom).

### Crystal data

**Table:**

C_18_H_18_N_2_O_4_S	*F*(000) = 752
*M**_r_* = 358.40	Prism
Monoclinic, *P*2_1_/*c*	*D*_x_ = 1.313 Mg m^−^^3^
Hall symbol: -P 2ybc	Melting point: 423 K
*a* = 17.3871 (7) Å	Mo *K*α radiation, λ = 0.71073 Å
*b* = 12.5318 (5) Å	θ = 0–25°
*c* = 8.4297 (3) Å	µ = 0.20 mm^−^^1^
β = 99.103 (2)°	*T* = 294 K
*V* = 1813.63 (12) Å^3^	Prism, colourless
*Z* = 4	0.36 × 0.28 × 0.22 mm

### Data collection

**Table:**

Bruker APEXII CCD diffractometer	3176 independent reflections
Radiation source: fine-focus sealed tube	2649 reflections with *I* > 2σ(*I*)
Graphite monochromator	*R*_int_ = 0.029
Detector resolution: 1.6 pixels mm^-1^	θ_max_ = 25.0°, θ_min_ = 1.2°
ω scans	*h* = −20→20
Absorption correction: multi-scan (*SADABS*; Bruker, 2009)	*k* = −14→14
*T*_min_ = 0.931, *T*_max_ = 0.957	*l* = −10→9
13884 measured reflections	

### Refinement

**Table:**

Refinement on *F*^2^	Primary atom site location: structure-invariant direct methods
Least-squares matrix: full	Secondary atom site location: difference Fourier map
*R*[*F*^2^ > 2σ(*F*^2^)] = 0.038	Hydrogen site location: inferred from neighbouring sites
*wR*(*F*^2^) = 0.113	H-atom parameters constrained
*S* = 1.07	*w* = 1/[σ^2^(*F*_o_^2^) + (0.0589*P*)^2^ + 0.3788*P*] where *P* = (*F*_o_^2^ + 2*F*_c_^2^)/3
3176 reflections	(Δ/σ)_max_ < 0.001
229 parameters	Δρ_max_ = 0.23 e Å^−^^3^
0 restraints	Δρ_min_ = −0.30 e Å^−^^3^
0 constraints	

### Special details

**Table:**

Geometry. All e.s.d.'s (except the e.s.d. in the dihedral angle between two l.s. planes) are estimated using the full covariance matrix. The cell e.s.d.'s are taken into account individually in the estimation of e.s.d.'s in distances, angles and torsion angles; correlations between e.s.d.'s in cell parameters are only used when they are defined by crystal symmetry. An approximate (isotropic) treatment of cell e.s.d.'s is used for estimating e.s.d.'s involving l.s. planes.
Refinement. Refinement of *F*^2^ against ALL reflections. The weighted *R*-factor *wR* and goodness of fit *S* are based on *F*^2^, conventional *R*-factors *R* are based on *F*, with *F* set to zero for negative *F*^2^. The threshold expression of *F*^2^ > σ(*F*^2^) is used only for calculating *R*-factors(gt) *etc*. and is not relevant to the choice of reflections for refinement. *R*-factors based on *F*^2^ are statistically about twice as large as those based on *F*, and *R*- factors based on ALL data will be even larger.

### Fractional atomic coordinates and isotropic or equivalent isotropic displacement parameters (Å^2^)

**Table:**

	*x*	*y*	*z*	*U*_iso_*/*U*_eq_	
C7	−0.33192 (14)	0.7433 (2)	0.5018 (3)	0.0863 (7)	
C16	0.44601 (18)	0.7075 (4)	0.2350 (6)	0.170 (2)	
H16A	0.4171	0.7677	0.1870	0.254*	
H16B	0.4942	0.7020	0.1936	0.254*	
H16C	0.4567	0.7168	0.3494	0.254*	
C18	0.4351 (2)	0.5073 (5)	0.2805 (5)	0.183 (2)	
H18A	0.4449	0.5197	0.3943	0.274*	
H18B	0.4833	0.4916	0.2435	0.274*	
H18C	0.4001	0.4481	0.2575	0.274*	
C13	0.19857 (10)	0.66538 (18)	0.2825 (2)	0.0598 (5)	
H13A	0.1805	0.7282	0.3330	0.072*	
H13B	0.2073	0.6087	0.3617	0.072*	
N1	−0.38426 (14)	0.7818 (2)	0.5450 (4)	0.1214 (9)	
C1	−0.13164 (10)	0.70509 (14)	0.4150 (2)	0.0508 (4)	
H1	−0.0852	0.7431	0.4249	0.061*	
C2	−0.19560 (11)	0.75155 (16)	0.4633 (2)	0.0587 (5)	
H2	−0.1925	0.8205	0.5048	0.070*	
C3	−0.26510 (11)	0.69460 (18)	0.4497 (3)	0.0650 (5)	
C4	−0.26913 (12)	0.5924 (2)	0.3865 (3)	0.0738 (6)	
H4	−0.3155	0.5543	0.3773	0.089*	
C5	−0.20474 (11)	0.54709 (16)	0.3371 (3)	0.0618 (5)	
H5	−0.2081	0.4787	0.2937	0.074*	
C6	−0.13473 (10)	0.60278 (14)	0.3517 (2)	0.0465 (4)	
C8	−0.06428 (10)	0.55338 (13)	0.3044 (2)	0.0448 (4)	
C9	−0.05133 (11)	0.44394 (14)	0.3242 (2)	0.0540 (5)	
H9	−0.0891	0.4041	0.3644	0.065*	
C10	0.06362 (11)	0.45021 (15)	0.2309 (2)	0.0573 (5)	
H10	0.1078	0.4160	0.2065	0.069*	
C11	0.05613 (10)	0.55892 (13)	0.2040 (2)	0.0465 (4)	
C12	−0.00844 (9)	0.61241 (13)	0.24214 (19)	0.0438 (4)	
H12	−0.0141	0.6856	0.2264	0.053*	
C14	0.27308 (11)	0.6900 (2)	0.2182 (3)	0.0650 (5)	
C15	0.39872 (15)	0.6065 (3)	0.1954 (3)	0.1037 (10)	
C17	0.37939 (19)	0.5867 (3)	0.0188 (4)	0.1206 (12)	
H17A	0.3463	0.5252	−0.0002	0.181*	
H17B	0.4265	0.5743	−0.0244	0.181*	
H17C	0.3530	0.6477	−0.0326	0.181*	
N2	0.01068 (10)	0.39231 (12)	0.2900 (2)	0.0609 (4)	
O4	0.27975 (10)	0.76620 (15)	0.1362 (2)	0.0938 (5)	
O3	0.32424 (8)	0.61363 (14)	0.26226 (18)	0.0771 (5)	
O1	0.09398 (8)	0.71741 (11)	0.03461 (16)	0.0627 (4)	
O2	0.16443 (8)	0.54780 (12)	0.02716 (17)	0.0701 (4)	
S1	0.12816 (2)	0.62493 (4)	0.11611 (5)	0.04867 (17)	

### Atomic displacement parameters (Å^2^)

**Table:**

	*U*^11^	*U*^22^	*U*^33^	*U*^12^	*U*^13^	*U*^23^
C7	0.0629 (14)	0.1074 (19)	0.0937 (18)	−0.0008 (13)	0.0278 (13)	−0.0104 (15)
C16	0.0595 (17)	0.286 (6)	0.172 (4)	−0.048 (3)	0.044 (2)	−0.076 (4)
C18	0.125 (3)	0.295 (6)	0.133 (3)	0.124 (4)	0.034 (2)	0.053 (4)
C13	0.0522 (10)	0.0798 (13)	0.0471 (10)	−0.0020 (10)	0.0066 (8)	−0.0097 (10)
N1	0.0791 (15)	0.152 (2)	0.143 (2)	0.0081 (15)	0.0480 (16)	−0.0270 (19)
C1	0.0500 (10)	0.0471 (9)	0.0554 (11)	−0.0024 (8)	0.0084 (8)	0.0019 (8)
C2	0.0620 (12)	0.0573 (11)	0.0580 (11)	0.0028 (9)	0.0136 (9)	−0.0043 (9)
C3	0.0557 (11)	0.0787 (14)	0.0633 (12)	0.0010 (10)	0.0175 (9)	−0.0019 (11)
C4	0.0544 (12)	0.0871 (15)	0.0825 (15)	−0.0177 (11)	0.0185 (11)	−0.0093 (13)
C5	0.0583 (11)	0.0599 (11)	0.0679 (13)	−0.0118 (9)	0.0123 (10)	−0.0093 (10)
C6	0.0498 (10)	0.0466 (9)	0.0422 (9)	−0.0035 (7)	0.0048 (7)	0.0039 (7)
C8	0.0494 (9)	0.0417 (9)	0.0404 (9)	−0.0025 (7)	−0.0022 (7)	−0.0024 (7)
C9	0.0607 (11)	0.0433 (9)	0.0548 (11)	−0.0050 (8)	−0.0009 (9)	−0.0002 (8)
C10	0.0588 (11)	0.0461 (10)	0.0645 (12)	0.0088 (8)	0.0021 (9)	−0.0083 (9)
C11	0.0489 (9)	0.0438 (9)	0.0450 (9)	0.0031 (7)	0.0016 (7)	−0.0060 (7)
C12	0.0504 (9)	0.0367 (8)	0.0428 (9)	0.0014 (7)	0.0030 (7)	−0.0020 (7)
C14	0.0531 (11)	0.0853 (15)	0.0557 (12)	−0.0063 (11)	0.0060 (9)	−0.0034 (11)
C15	0.0592 (14)	0.178 (3)	0.0767 (17)	0.0234 (17)	0.0182 (13)	−0.0039 (18)
C17	0.111 (2)	0.173 (3)	0.085 (2)	0.028 (2)	0.0357 (18)	−0.008 (2)
N2	0.0655 (10)	0.0415 (8)	0.0720 (11)	0.0035 (7)	−0.0003 (9)	−0.0032 (7)
O4	0.0804 (11)	0.0995 (13)	0.1034 (14)	−0.0137 (9)	0.0205 (10)	0.0215 (11)
O3	0.0532 (8)	0.1156 (13)	0.0628 (9)	0.0117 (8)	0.0100 (7)	0.0074 (8)
O1	0.0598 (8)	0.0680 (8)	0.0619 (8)	0.0062 (6)	0.0144 (6)	0.0128 (7)
O2	0.0682 (9)	0.0816 (10)	0.0631 (8)	0.0095 (7)	0.0183 (7)	−0.0248 (7)
S1	0.0487 (3)	0.0559 (3)	0.0418 (3)	0.00573 (19)	0.00817 (18)	−0.00699 (19)

### Geometric parameters (Å, º)

**Table:**

C7—N1	1.139 (3)	C5—H5	0.9300
C7—C3	1.441 (3)	C6—C8	1.482 (2)
C16—C15	1.518 (5)	C8—C12	1.388 (2)
C16—H16A	0.9600	C8—C9	1.396 (2)
C16—H16B	0.9600	C9—N2	1.327 (2)
C16—H16C	0.9600	C9—H9	0.9300
C18—C15	1.521 (5)	C10—N2	1.329 (2)
C18—H18A	0.9600	C10—C11	1.384 (3)
C18—H18B	0.9600	C10—H10	0.9300
C18—H18C	0.9600	C11—C12	1.388 (2)
C13—C14	1.513 (3)	C11—S1	1.7595 (18)
C13—S1	1.7830 (18)	C12—H12	0.9300
C13—H13A	0.9700	C14—O4	1.195 (3)
C13—H13B	0.9700	C14—O4	1.195 (3)
C1—C2	1.373 (3)	C14—O4	1.195 (3)
C1—C6	1.386 (2)	C14—O3	1.320 (3)
C1—H1	0.9300	C15—O3	1.494 (3)
C2—C3	1.392 (3)	C15—C17	1.495 (4)
C2—H2	0.9300	C17—H17A	0.9600
C3—C4	1.385 (3)	C17—H17B	0.9600
C4—C5	1.377 (3)	C17—H17C	0.9600
C4—H4	0.9300	O1—S1	1.4280 (14)
C5—C6	1.392 (2)	O2—S1	1.4297 (13)
			
N1—C7—C3	179.1 (3)	N2—C9—C8	125.05 (18)
C15—C16—H16A	109.5	N2—C9—H9	117.5
C15—C16—H16B	109.5	C8—C9—H9	117.5
H16A—C16—H16B	109.5	N2—C10—C11	123.10 (17)
C15—C16—H16C	109.5	N2—C10—H10	118.5
H16A—C16—H16C	109.5	C11—C10—H10	118.5
H16B—C16—H16C	109.5	C10—C11—C12	119.73 (17)
C15—C18—H18A	109.5	C10—C11—S1	118.50 (14)
C15—C18—H18B	109.5	C12—C11—S1	121.74 (13)
H18A—C18—H18B	109.5	C8—C12—C11	118.02 (15)
C15—C18—H18C	109.5	C8—C12—H12	121.0
H18A—C18—H18C	109.5	C11—C12—H12	121.0
H18B—C18—H18C	109.5	O4—C14—O3	128.3 (2)
C14—C13—S1	107.26 (13)	O4—C14—O3	128.3 (2)
C14—C13—H13A	110.3	O4—C14—O3	128.3 (2)
S1—C13—H13A	110.3	O4—C14—C13	122.5 (2)
C14—C13—H13B	110.3	O4—C14—C13	122.5 (2)
S1—C13—H13B	110.3	O4—C14—C13	122.5 (2)
H13A—C13—H13B	108.5	O3—C14—C13	109.17 (19)
C2—C1—C6	121.49 (17)	O3—C15—C17	108.3 (2)
C2—C1—H1	119.3	O3—C15—C16	109.8 (2)
C6—C1—H1	119.3	C17—C15—C16	112.7 (3)
C1—C2—C3	119.43 (19)	O3—C15—C18	101.1 (3)
C1—C2—H2	120.3	C17—C15—C18	110.2 (3)
C3—C2—H2	120.3	C16—C15—C18	114.0 (3)
C4—C3—C2	119.74 (19)	C15—C17—H17A	109.5
C4—C3—C7	121.0 (2)	C15—C17—H17B	109.5
C2—C3—C7	119.3 (2)	H17A—C17—H17B	109.5
C5—C4—C3	120.25 (19)	C15—C17—H17C	109.5
C5—C4—H4	119.9	H17A—C17—H17C	109.5
C3—C4—H4	119.9	H17B—C17—H17C	109.5
C4—C5—C6	120.53 (19)	C9—N2—C10	116.72 (16)
C4—C5—H5	119.7	C14—O3—C15	121.6 (2)
C6—C5—H5	119.7	O1—S1—O2	118.74 (9)
C1—C6—C5	118.55 (17)	O1—S1—C11	108.33 (8)
C1—C6—C8	120.42 (15)	O2—S1—C11	107.78 (9)
C5—C6—C8	121.01 (16)	O1—S1—C13	109.23 (10)
C12—C8—C9	117.37 (16)	O2—S1—C13	107.45 (9)
C12—C8—C6	122.44 (15)	C11—S1—C13	104.38 (9)
C9—C8—C6	120.19 (16)		
			
C6—C1—C2—C3	0.4 (3)	S1—C13—C14—O3	−107.14 (17)
C1—C2—C3—C4	−0.6 (3)	C8—C9—N2—C10	−0.7 (3)
C1—C2—C3—C7	179.5 (2)	C11—C10—N2—C9	−0.4 (3)
C2—C3—C4—C5	0.0 (3)	O4—C14—O4—O4	0.0 (4)
C7—C3—C4—C5	179.9 (2)	O3—C14—O4—O4	0.0 (4)
C3—C4—C5—C6	0.7 (3)	C13—C14—O4—O4	0.0 (3)
C2—C1—C6—C5	0.2 (3)	O4—C14—O4—O4	0.0 (4)
C2—C1—C6—C8	−178.38 (17)	O3—C14—O4—O4	0.0 (4)
C4—C5—C6—C1	−0.8 (3)	C13—C14—O4—O4	0.0 (3)
C4—C5—C6—C8	177.81 (19)	O4—C14—O3—C15	−7.3 (4)
C1—C6—C8—C12	−33.7 (2)	O4—C14—O3—C15	−7.3 (4)
C5—C6—C8—C12	147.77 (18)	O4—C14—O3—C15	−7.3 (4)
C1—C6—C8—C9	145.27 (18)	C13—C14—O3—C15	170.12 (19)
C5—C6—C8—C9	−33.3 (2)	C17—C15—O3—C14	−63.6 (4)
C12—C8—C9—N2	0.9 (3)	C16—C15—O3—C14	59.8 (3)
C6—C8—C9—N2	−178.05 (16)	C18—C15—O3—C14	−179.4 (3)
N2—C10—C11—C12	1.2 (3)	C10—C11—S1—O1	153.88 (15)
N2—C10—C11—S1	−176.94 (15)	C12—C11—S1—O1	−24.24 (16)
C9—C8—C12—C11	0.0 (2)	C10—C11—S1—O2	24.22 (17)
C6—C8—C12—C11	178.94 (15)	C12—C11—S1—O2	−153.90 (14)
C10—C11—C12—C8	−1.0 (2)	C10—C11—S1—C13	−89.82 (16)
S1—C11—C12—C8	177.12 (12)	C12—C11—S1—C13	92.06 (15)
S1—C13—C14—O4	70.4 (3)	C14—C13—S1—O1	−82.29 (16)
S1—C13—C14—O4	70.4 (3)	C14—C13—S1—O2	47.76 (18)
S1—C13—C14—O4	70.4 (3)	C14—C13—S1—C11	162.04 (15)

### Hydrogen-bond geometry (Å, º)

**Table:**

*D*—H···*A*	*D*—H	H···*A*	*D*···*A*	*D*—H···*A*
C9—H9···O1^i^	0.93	2.50	3.210 (2)	134
C12—H12···N2^ii^	0.93	2.60	3.518 (2)	172
C13—H13*A*···O1^iii^	0.97	2.54	3.347 (2)	141
C16—H16*A*···O4	0.96	2.36	2.971 (4)	121

Symmetry codes: (i) −*x*, *y*−1/2, −*z*+1/2; (ii) −*x*, *y*+1/2, −*z*+1/2; (iii) *x*, −*y*+3/2, *z*+1/2.
